# Paracrine and epigenetic control of CAF-induced metastasis: the role of HOTAIR stimulated by TGF-ß1 secretion

**DOI:** 10.1186/s12943-018-0758-4

**Published:** 2018-01-11

**Authors:** Yu Ren, Huan-huan Jia, Yi-qi Xu, Xuan Zhou, Xiao-hui Zhao, Yun-fei Wang, Xin Song, Zhi-yan Zhu, Ting Sun, Yan Dou, Wei-ping Tian, Xiu-lan Zhao, Chun-sheng Kang, Mei Mei

**Affiliations:** 1 Research Center of Basic Medical Sciences, Tianjin Medical University, Tianjin, 300070 China; 2 Department of Head and Neck, Tianjin Medical University Cancer Hospital, Tianjin, 300060 China; 3 Department of Obstetrics and Gynecology, Tianjin Medical University General Hospital, Tianjin, 300052 China; 4 Department of Neurosurgery, Tianjin Medical University General Hospital, Lab of Neuro- oncology, Tianjin Neurological Institute, Key Laboratory of Post-Neuroinjury Neuro-repair and Regeneration in Central Nervous System, Ministry of Education and Tianjin City, Tianjin, 300052 China; 5 Department of Pathology, Tianjin Medical University, Tianjin, 300070 China

**Keywords:** Carcinoma associated fibroblasts, TGF-β1, HOTAIR, Epigenetic control, Metastasis

## Abstract

**Background:**

The communication between carcinoma associated fibroblasts (CAFs) and cancer cells facilitate tumor metastasis. In this study, we further underlying the epigenetic mechanisms of CAFs feed the cancer cells and the molecular mediators involved in these processes.

**Methods:**

MCF-7 and MDA-MB-231 cells were treated with CAFs culture conditioned medium, respectively. Cytokine antibody array, enzyme-linked immunosorbent assay, western blotting and immunofluorescence were used to identify the key chemokines. Chromatin immunoprecipitation and luciferase reporter assay were performed to explore the transactivation of target LncRNA by CAFs. A series of in vitro assays was performed with RNAi-mediated knockdown to elucidate the function of LncRNA. An orthotopic mouse model of MDA-MB-231 was conducted to confirm the mechanism in vivo.

**Results:**

Here we reported that TGF-β1 was top one highest level of cytokine secreted by CAFs as revealed by cytokine antibody array. Paracrine TGF-β1 was essential for CAFs induced EMT and metastasis in breast cancer cells, which is a crucial mediator of the interaction between stromal and cancer cells. CAF-CM significantly enhanced the HOTAIR expression to promote EMT, whereas treatment with small-molecule inhibitors of TGF-β1 attenuated the activation of HOTAIR. Most importantly, SMAD2/3/4 directly bound the promoter site of HOTAIR, located between nucleotides -386 and -398, -440 and -452, suggesting that HOTAIR was a directly transcriptional target of SMAD2/3/4. Additionally, CAFs mediated EMT by targeting CDK5 signaling through H3K27 tri-methylation. Depletion of HOTAIR inhibited CAFs-induced tumor growth and lung metastasis in MDA-MB-231 orthotopic animal model.

**Conclusions:**

Our findings demonstrated that CAFs promoted the metastatic activity of breast cancer cells by activating the transcription of HOTAIR via TGF-β1 secretion, supporting the pursuit of the TGF-β1/HOTAIR axis as a target in breast cancer treatment.

**Electronic supplementary material:**

The online version of this article (10.1186/s12943-018-0758-4) contains supplementary material, which is available to authorized users.

## Background

Breast cancer is the most malignant disease in women. Specifically, high rates of metastasis to the lymph nodes, lungs, bone and brain, not the primary tumor, are the leading cause of breast cancer death [1]. Therefore, improving our understanding of the molecular mechanisms of tumor metastasis may lead to more effective strategies for the prognosis and treatment of breast cancer.

Growing evidence indicates that malignant breast tissue requires complex local and systemic stromal interactions to provide a tumor-promoting environment during breast carcinoma development and progression [2, 3]. Specifically, tumor stromal cells cross-communicate and develop an aggressive phenotype of cancer cells, which are recognized as an important modulator and even a driver of tumorigenicity [4]. Cancer associated fibroblasts (CAFs), a key component of the tumor microenvironment, have been proven to be a major contributor of various processes, such as proliferation, invasion, angiogenesis and drug resistance [5–7]. These effects are mediated by paracrine stimulation from a variety of growth factors and cytokines, including transforming growth factor β1 (TGF-β1), basic fibroblast growth factor (b-FGF), vascular endothelial growth factor (VEGF), platelet-derived growth factor (PDGF), and interleukins (IL) [8, 9]. Our previous study indicated that CAFs stimulated epithelial-mesenchymal transition (EMT) and impaired taxol efficacy in breast cancer by elevating NF-κB/miR-21 signaling [10]. However, the epigenetic mechanisms by which CAFs feed the cancer cells and allow them to acquire an aggressive phenotype and the molecular mediators involved in these processes have not been extensively studied.

In addition to the several well-documented gene mutations that have been associated with the development of breast cancer, considerable attention is being focused on the participation of epigenetic events, including the diverse activities of non-coding RNAs [11]. Highly up-regulated in breast cancer, the lncRNA HOX transcript antisense RNA (HOTAIR) mediates H3K27 tri-methylation and the epigenetic silencing of tumor suppressor genes by recruiting enhancer of zeste homolog 2 (EZH2), which is considered a key molecule and potential biomarker for breast cancer [12]. Moreover, HOTAIR is reportedly involved in drug resistance and stemness maintenance in breast cancer cell lines [13–15]. Importantly, growing evidence indicates that HOTAIR promotes metastasis breast, pancreatic and hepatocellular carcinoma [16–19]. Given its critical role during tumor progression, HOTAIR is a novel target for breast cancer therapy.

The activation of CDK5 signaling has been implicated in the control of cell motility and metastatic potential, which are significantly correlated with several markers of poor prognosis in breast cancer [20–22]. Our previous study demonstrated that the aberrant activation of CDK5 signaling is associated with lymph node metastasis in breast cancer, which was responsible for high-dose taxol-induced invasion and EMT [23]. However, the mechanism underlying the activation of CDK5 remains elusive. Moreover, CDK5 was proven to be essential for TGF-β1-induced EMT in breast cancer progression [24]. Strikingly, aberrant CDK5 promoter DNA hypomethylation was identified in the mantle cell lymphoma genome compared with normal naive B cells [25]. These findings indicate an interaction between CDK5 signaling and tumor stromal cells, which may underlie the novel epigenetic mechanism of tumor environment-induced metastasis and hold therapeutic potential in breast cancer.

Based on these previous studies, we further demonstrated that CAFs promoted the metastasis of breast cancer cells via paracrine TGF-β1, which is a crucial mediator of the interaction between stromal and cancer cells. Importantly, CAFs transactivated HOTAIR to promote EMT. Strikingly, we identified HOTAIR as a direct transcriptional target of SMAD2/3/4. Additionally, CAFs mediated HOTAIR expression and EMT by targeting CDK5 signaling, and H3K27 trimethylation was highly enriched at the promoter of CDK5RAP1 and EGR-1. Overall, we describe the epigenetic mechanisms underlying the CAF-induced aggressive behavior of cancer cells, which support the targeting of the TGF-β1/ HOTAIR axis as a novel strategy for breast cancer treatment.

## Methods

### Cell culture

The human breast cancer cell lines MDA-MB-231 and MCF-7 were purchased from the American Type Culture Collection (ATCC). The CAFs were isolated and cultured as described previously [23]. CAFs were acquired from four invasive breast cancer patients who underwent a mastectomy at the Tianjin Medical University Cancer Institute and Hospital (TMUCIH), and the use of specimens was approved by the Institutional Review Board of TMUCIH. CAFs were obtained from tissues that had been cut into small pieces and digested with collagenase type I (1 mg/mL; Sigma) and hyaluronidase (125 units/mL; Sigma) for 6 h in DMEM without FBS at 37 °C. After filtering the undigested tissues, the stromal fraction was centrifuged at 1000 rpm for 5 min. The cells were cultured in Dulbecco’s Modified Eagle’s Medium (DMEM) or RMPI Medium 1640 supplemented with 10% fetal bovine serum (FBS), 1% penicillin and 1% streptomycin at 37 °C in a 5% CO_2_ humidified incubator.

### Cytokine antibody array

The profiles of cytokines secreted by CAFs were detected in the culture supernatants using a Human Cytokine Array (RayBiotech, Guangzhou, China) according to the manufacturer’s instructions. The cytokines with distinct differences in expression were screened out.

### Enzyme-linked immunosorbent assay (ELISA)

The culture medium was removed and used to assess the level of extracellular TGF-β1 with a TGF-β1 enzyme-linked immunosorbent assay (ELISA, abcam) according to the manufacturer’s instructions. The results are expressed in ng/mL.

### Western blotting

The total protein was extracted with cold radio-immunoprecipitation buffer containing protease inhibitor and phosphatase inhibitor. The cell lysates were separated using 8-10% SDS–PAGE and transferred onto polyvinylidene difluoride (PVDF) membranes. Antibodies against human CDK5, CDK5RAP1, H3K27me3, EZH2 (1:1000 dilutions, Cell Signaling Technology), Egr-1, E-cadherin, vimentin, β-catenin (1:1000 dilutions, Abcam), and β-actin (1:4000 dilutions, Santa Cruz) were used as primary antibodies. Rabbit or mouse IgG antibody coupled with horseradish peroxidase (Santa Cruz) was used as a secondary antibody. The antibody-labeled protein bands on membranes were detected with a G-BOX iChemi XT instrument (Syngene).

### Immunofluorescence

After various treatments, cells were seeded on sterile coverslips and cultured for 48 h. The cells were then fixed with 4% paraformaldehyde for 15 min and permeabilized with 0.25% Triton-X 100 for 10 min, followed by blocking with 3% BSA for 1 h. Immunofluorescence staining was conducted with antibodies against β-catenin, E-cadherin and vimentin (1:100 dilutions, Abcam). The cells were washed with phosphate-buffered saline (PBS) and incubated with Alexa Fluor 488 or Alexa Fluor 546 (Life Technologies) secondary antibodies. To detect the formation of stress fibers, cells were stained with Alexa Fluor 568-phalloidin. Nuclei were stained using DAPI, and the cells were visualized using FV-1000 laser scanning confocal microscopes. Cells from three independent experiments in which at least 300 cells were counted were quantified.

### Quantitative real-time PCR

Total RNA was extracted using TRIzol reagent (Life Technologies) according to the standard protocol. HOTAIR cDNA was obtained from total RNA using the Fast Quant RT Kit (TIANGEN). Real-time PCR was performed using Super Real Pre Mix Plus SYBR Green (TIANGEN) on an ABI-7500 Real-time PCR machine (Life Technologies).The following thermocycling protocol was employed: 95 °C for 30s, 60 °C for 30 s and 72 °C for 30 s for 40 cycles. GAPDH was used as the internal control. Data are presented as fold changes (2^−ΔΔCt^) and were analyzed using the Opticon Monitor Analysis Software V 2.02 (MJ Research).

### Wound-healing assay

Approximately 2 × 10^5^ MDA-MB-231 cells or 3 × 10^5^ MCF-7 cells were plated in six-well plates after different treatments. A linear scratch/wound was made on the cell monolayer with a sterile pipette. Photomicrographs of live cells were taken at 40× magnification, and the distance migrated was observed after 48 h or 72 h.

### Matrigel invasion assay

The Matrigel invasion assay was performed in 24-well Transwell culture plates. Briefly, 40 μL of Matrigel (1 mg/mL, BD) was applied to 8 μm polycarbonate membrane filters. Approximately 3 × 10^4^ MDA-MB-231 cells or 5 × 10^4^ MCF-7 cells were resuspended and then seeded in 24-well Transwell plates containing FBS-free medium in the upper chamber and complete growth medium supplemented with 10% FBS in the lower chamber for 24 h or 48 h at 37 °C. Non-invading cells were removed from the upper surfaces of the invasion membranes, and the cells on the lower surface were stained with hematoxylin. The average number of cells per field was determined by counting the cells in six random fields per well. Cells were counted in four separate fields in three independent experiments.

### Fluorescence in situ hybridization (FISH)

The expression of HOTAIR in paraffin-embedded sections and cells of different conditions seeded on the sterile coverslips for 48 h was examined in situ hybridization. First, we deparaffinized and rehydrated the samples. Before digesting samples with proteinase K, we used 4% paraformaldehyde to fix the paraffin-embedded sections and DEPC water to fix the cell samples. We then hybridized samples with a 5’Cy3-labeled modified HOTAIR probe (6 ng/μl) overnight at 37 °C. After completing the hybridization, the samples were successively washed twice with solution buffer (2×, 1×, 0.5×, chloride sodium citrate buffer) at 37 °C for 10 min each. Nuclei were stained using DAPI, and the cells were visualized using an FV-1000 laser scanning confocal microscope.

### Chromatin immunoprecipitation assay (ChIP)

The EZ-ChIP kit (Millipore) was used to perform the ChIP assays. Briefly, 1% formaldehyde was used to fix cells, and 0.125 M glycine was then used to neutralize the formaldehyde. The cells were then lysed in lysis buffer with SDS and protease inhibitors. Soluble chromatins of 200-1000 bp were collected after sonication and pre-cleared in 1:10 dilution buffer, followed by incubation with IgG, H3K27me3 and EZH2 anti-bodies on a rotating platform overnight at 4 °C. After washing the immunocomplexes captured by protein G-Sepharose beads, the bound DNA fragments were eluted for real-time qPCR analysis using ChIP primer.

### Luciferase reporter assay

The upstream 2.5 kb promoter regions of HOTAIR were cloned into a luciferase reporter vector (GV238 vector), and the mutations of predicted SMADs binding sites were also cloned into the same luciferase reporter vector. Cells were co-transfected with SMAD plasmid GV219 vector and HOTAIR plasmid (wild type or mutant) for 24 h, and the promoter activities were examined with a luciferase assay.

### Orthotopic nude mouse models and treatment

BALB/c nude mice aged 4-6 weeks were purchased from the Animal Center at the Cancer Institute at Chinese Academy of Medical Science (Beijing, China). MDA-MB-231 cells transfected with Lenti-HOTAIR or Lenti NC and CAFs were injected into the mammary fat pads of each nude mouse at a ratio of 1:3. The mice were imaged for luciferase activity with an IVIS imaging system once per week.

### Hematoxylin and eosin staining and immunohistochemistry analysis

The paraffin-embedded tissue sections were stained with hematoxylin and eosin (H&E) and subjected to an immunohistochemistry analysis. After blocking with 3% H_2_O_2_ and non-immune rabbit serum, the sections were incubated with primary antibodies (1:200 dilutions) overnight at 4 °C, followed by a streptavidin-biotinylated secondary antibody for 1 h at 37 °C. The chromogenic substrate was developed with 3, 3′-diaminobenzidine, andhematoxylin was used for counter staining. The results were visualized using a light microscope.

### Statistical analyses

SPSS 16.0 (IBM, USA) was used for all calculations. All values are presented as the mean ± SD. Statistical comparisons between the two groups were made using Student’s t-test, and differences among groups were assessed with a two-way ANOVA, followed by Dunnett’s test. Significance was set to *P* < 0.05.*P < 0.05, **P < 0.01.

## Results

### Paracrine TGF-β1 was essential for CAFs to promote the metastasis of breast cancer cells

**Fig. 1:**
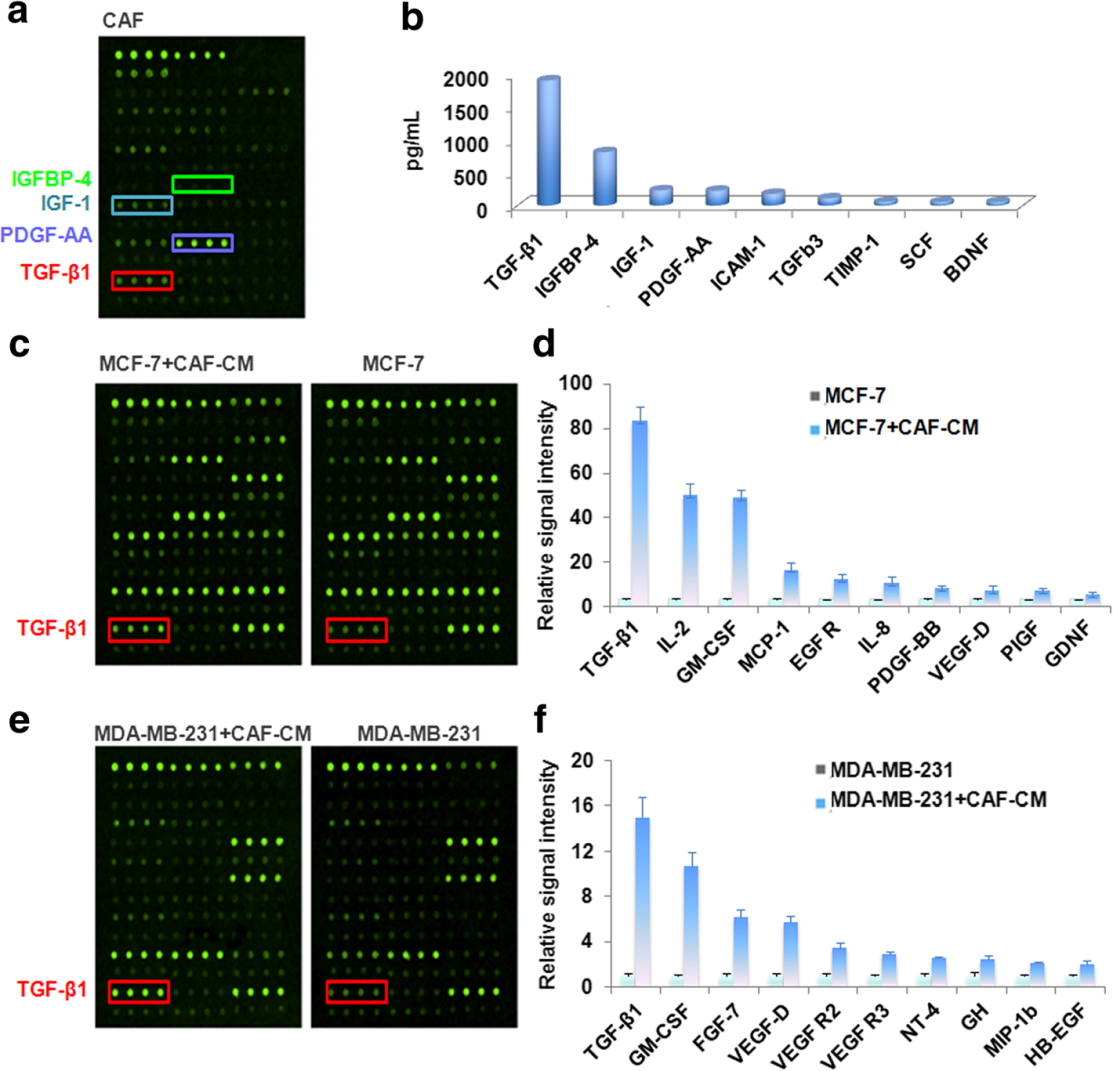
TGF-β1 was the biggest increased cytokine in CAF-CM treated cells. **a** Cytokine antibody array of culture medium of CAFs. **b** The top ten cytokine secreted by CAFs. Cytokine antibody array of culture medium of MCF-7 (**c**), MDA-MB-231(**e**) and by treatment of CAF-CM. Relative signal intensity of top ten increased cytokine in CAF-CM treated MCF-7 (**d**), MDA-MB-231(**f**), compared to the control cells

During breast cancer progression, CAFs may supply appropriate signals that may promote the development of aggressive phenotypes in carcinoma cells and establish a complex scenario, which culminates in metastasis [26]. To identify the cytokines secreted by CAFs that enhance tumorigenic ability, the cytokine profiles of cell supernatants from MCF-7, MDA-MB-231 and treatment with conditioned media of CAFs (CAF-CM) were analyzed using a RayBiotech Human Cytokine Antibody Array. The top four cytokines secreted in the supernatant of CAFs were TGF-β1, IGFBP-4, IGF-1 and PDGF-AA (Fig. 1a, b). Furthermore, TGF-β1 was the biggest increased cytokine in both CAF-CM treated MCF-7 (Fig. 1c, d) as well as in MDA-MB-231 cells, compared to control cells (Fig.1e, f).

To gain a further insight of the role of CAFs in the clinical setting, the distribution of TGF-ß1 in clinic samples was performed (Additional file 1: Figure S1). It’s well documented that CAFs strongly expressed α-SMA, whereas were negative for the epithelial marker cytokeratin. Immunofluorescent staining with antibodies against α-SMA, cytokeratin-7 and TGF-ß1 showed that the red fluorescence of TGF-ß1 colocalized with green fluorescence of α-SMA, but not with cytokeratin-7 in both invasive breast carcinoma and ductal carcinoma in situ. More important, much more and stronger fluorescence of TGF-ß1 was detected in invasive breast carcinoma, compared with human ductal carcinoma in situ. Collectively, these results indicated that TGF-ß1may be secreted by CAFs.

**Fig. 2:**
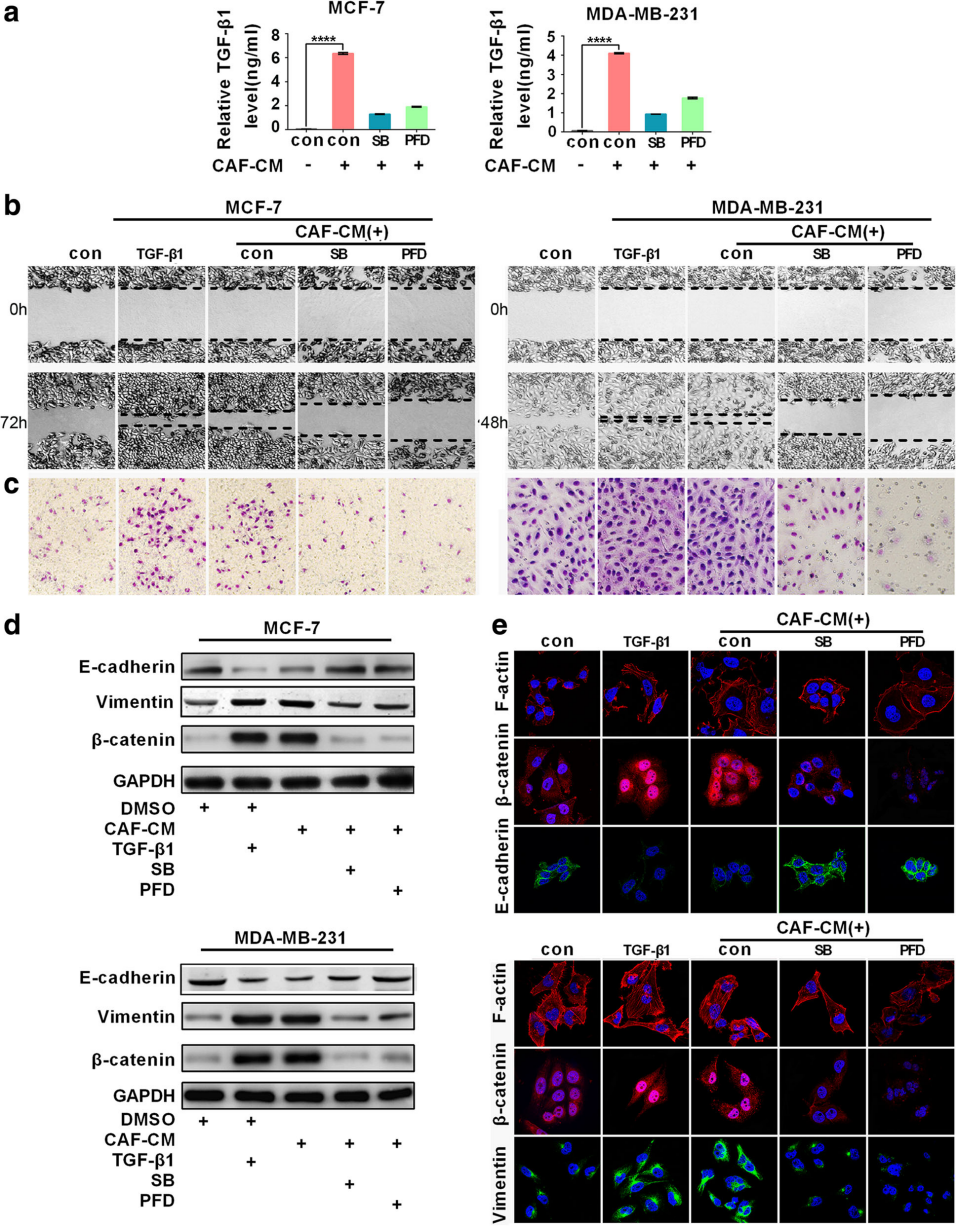
Paracrine TGF-β1 was enssential for CAFs induced EMT. **a** The amount of TGF-β1 released into the cell culture supernatant of cells treated with CAF-CM was determined by ELISA. **b** The wound-healing assay of MCF-7 and MDA-MB-231 cells treated with recombinant TGF-β1, CAF-CM, the TGF-β1 inhibitor SB431542 (SB) or pirfenidone (PFD), respectively. **c** SB or PFD treatment impaired the CAF-mediated increase in invading cancer cells, as indicated by the Transwell assay. **d** Western blotting analysis for E-cadherin, vimentin and β-catenin of cells treated as in (**b**). **e** Immunofluorescence staining showed that treatment with SB or PFD increased E-cadherin levels, decreased vimentin and nuclear β-catenin distribution in MCF-7 and MDA-MB-231 cells. CAFs promoted F-actin polymerization and stability in cancer cells, whereas treatment with SB or PFD impaired the formation of stress fibers. Scale bar: 15 μm

Due to the changes of quantity and quality in CAFs from different patients, we extracted CAFs from four invasive breast carcinomas patients to reduce the original difference in this study (Additional file 1: Figure S2). MDA-MB-231 and MCF-7 cells were treated with conditioned media of CAF1#, CAF2#, CAF3# and CAF4#, respectively. ELISA further confirmed all of the four CAFs populations induced significant increased TGF-β1 level in the supernatants from CAF-CM-treated cells (mean value: 0.05 ± 0.01 ng/ml vs 6.34 ± 0.243 ng/ml in MCF-7 cells; 0.06 ± 0.01 ng/ml vs 4.10 ± 0.14 ng/ml in MDA-MB-231 cells) (Fig. 2a, Additional file 1: Figure S3). We then further evaluated the role of TGF-β1 in CAFs induced EMT of cancer cells. We found that TGF-β1 treatment achieved similar enhanced the migratory activity of cancer cell, compared with CAF-CM treated cells (Fig. 2b). Additionally, the small-molecule inhibitors of TGF-β1, SB451332 (SB) or Pirfenidone (PFD) treatment abrogated the CAF-CM induced promotion of cell migration. Moreover, all of the four CAFs populations were competent in enhancing the cell invasive ability, while the number of invading cells was deceased 2- to 3.5-fold in SB or PFD treated cells, as indicated by a Transwell assay (Fig. 2c, Additional file 1: Figure S4). Moreover, SB and PDF treatment markedly rescued the induction of vimentin and β-catenin expression as well as the repression of E-cadherin in CAF-CM-treated cells (Fig. 2d). Immunofluorescent images showed that SB or PFD treatment decreased the nuclear signals of ß-catenin in MDA-MB-231 and MCF-7 cells, suggesting that the nuclear translocation to the cytoplasm was promoted (Fig. 2e). Furthermore, the remodeling and polarization of F-actin was observed after CAF stimulation, whereas the formation of a stress fiber pattern was abolished after SB or PFD treatment. These results identified TGF-β1 as a crucial mediator of the interaction between CAFs and breast cancer cells and TGF-β1 was necessary for CAFs to induce EMT of cancer cells.

### CAFs induced EMT by activating the expression of lncRNA HOTAIR in breast cancer cells

**Fig. 3:**
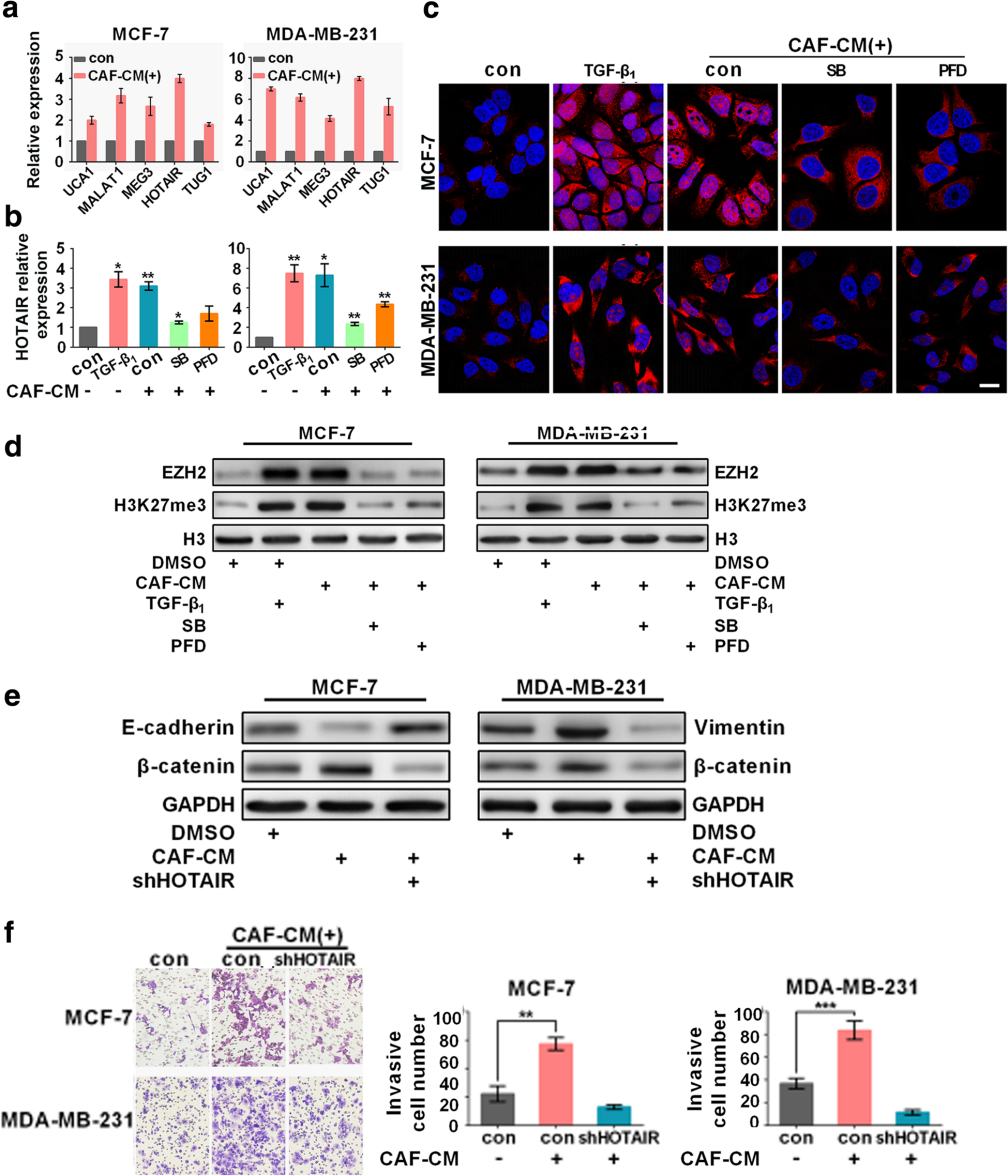
CAFs enhanced the expression of the lncRNA HOTAIR in breast cancer cells to induce EMT. **a** The expression of seveal metastasis associated LncRNAs treated with CAF-CM was measured by RC-PCR. HOTAIR levels in MDA-MB-231 and MCF-7 cells upon treatment with recombinant TGF-β1, SB or PFD was measured by real-time RT-PCR (**b**) or FISH (**c**). Scale bar: 15 μm. **d** Western blotting indicated that the EZH2 and H3K27me3 expression levels were significantly increased in CAF-CM-treated cells. **e** The expression levels of the indicated epithelial or mesenchymal markers in MDA-MB-231 and MCF-7 cells were measured by western blotting. **f** The depletion of HOTAIR decreased the number of invaded cells, as indicated by the Transwell assay. Quantitation of invasive MCF-7 and MDA-MB-231 cells. For each measurement, ≥ 10 fields of view with ≥ 100 cells were analyzed; *P < 0.05, **P < 0.01

The epigenetic regulation of lncRNA triggered invasion and metastasis in breast cancer. To study the epigenetic mechanism by which CAFs induce EMT in cancer cells, the expression of several lncRNAs were measured by RT-PCR (Fig. 3a). Specifically, CAF-CM resulted in the largest increases in HOTAIR expression, 3.5- and 8.7-fold increases inMCF-7 and MDA-MB-231 cells, respectively, compared to the control cells. Similar to CAF-CM, recombinant TGF-β1 also increased the levels of HOTAIR (Fig. 3b). However, the CAF-CM mediated activation of HOTAIR was attenuated after SB or PFD treatment. FISH images revealed that SB or PFD repressed the nuclear distribution of HOTAIR induced by CAF-CM (Fig. 3c), and Western blotting analysis indicated that the protein levels of EZH2 and tri-methylated H3K27 were notably increased (Fig. 3d). Moreover, the induction of E-cadherin and repression of vimentin and β-catenin was observed in HOTAIR knock-down cells (Fig. 3e), and the depletion of HOTAIR decreased the number of invading cells, as indicated by a Transwell assay (Fig. 3f, Additional file 1: Figure S5). Based on these data, we concluded that CAFs promoted metastasis by activating HOTAIR expression in cancer cells. However, the mechanisms by which CAFs activate HOTAIR remain to be addressed.

### HOTAIR is a transcriptional target of SMAD2/3/4

**Fig. 4:**
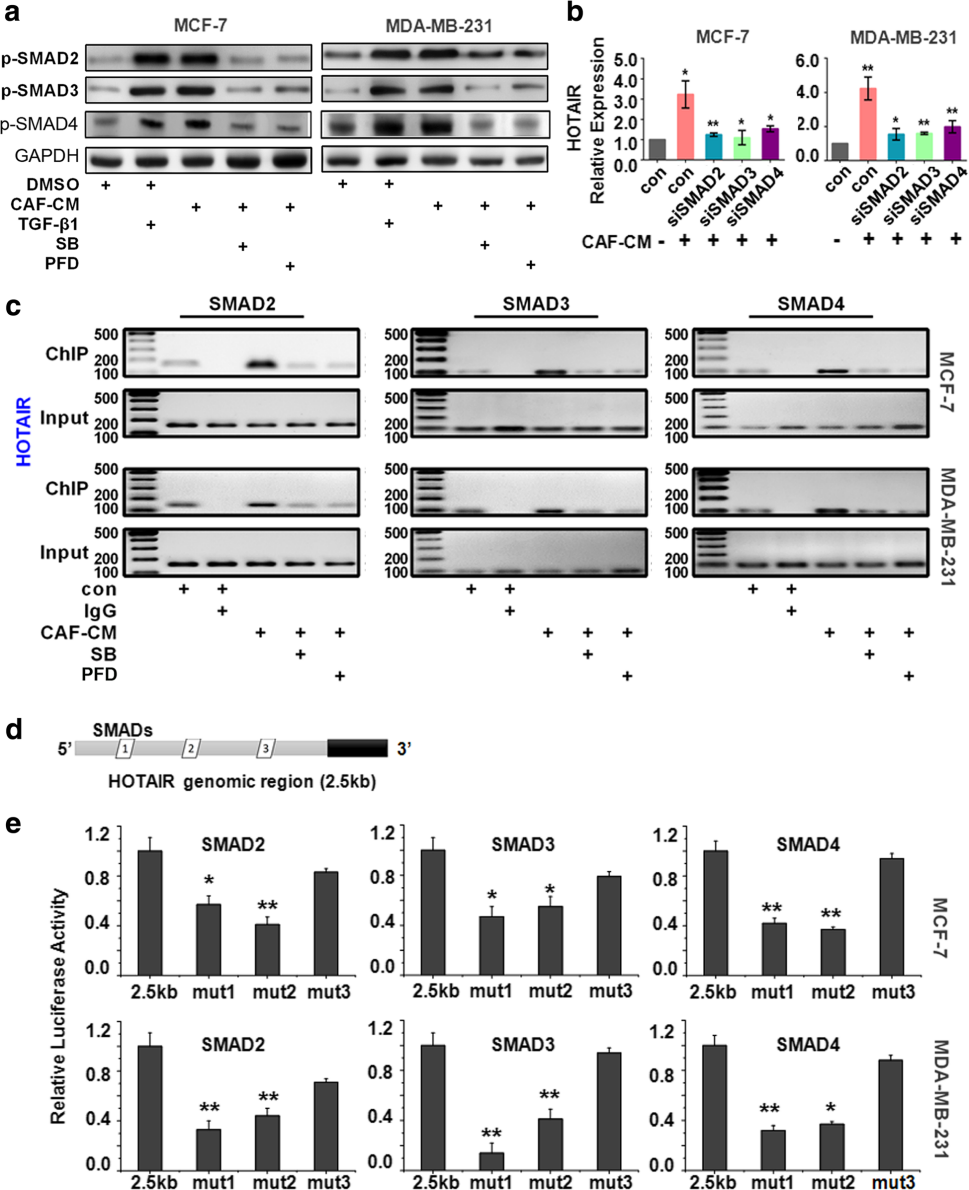
CAFs activated the transcription of HOTAIR by SMAD2/3/4. **a** Western blotting analysis of the effect of recombinant TGF-β1, CAF-CM, SB or PFD treatment on the p-SMAD2, p-SMAD3 and p-SMAD4 in MCF-7 and MDA-MB-231 cells. **b** The HOTAIR expression upon treatment of siRNA against SMAD2, SMAD3, and SMAD4 was determined by real time PCR. **c** SMAD2/3/4 were enriched in the promoter of HOTAIR in CAF-CM-treated MDA-MB-231 and MCF-7 cells, as determined by a CHIP assay. **d** A bioinformatics assay showed the predicted binding sites of SMAD2/3/4 within the HOTAIR promoter region. **e** A mutational analysis of HOTAIR promoter activity. The wild-type and mutant promoter constructs were co-transfected with SMAD2, 3, or 4 plasmids into MCF-7 and MDA-MB-231 cells. The promoter activity was determined by a luciferase assay

TGF-β is known to assemble a receptor complex that activates Smads thereby assembling multi-subunit complexes that regulate transcription [27]. Western blotting analysis indicated that the levels of phosphorylated of Smad2 protein (p-SMAD2), p-SMAD3 and p-SMAD4were significantly elevated in CAF-CM and TGF-β1-treated cancer cells, suggesting that CAFs activated the TGF-β1/SMAD signaling pathway (Fig. 4a). However, the repression of p-SMAD2, p-SMAD3 and p-SMAD4 was detected in cells treated with SB or PFD. Moreover, RT-PCR showed a 50-60% reduction in HOTAIR expression in cells transfected with SMAD2, SMAD3 or SMAD4 siRNA (Fig. 4b). To further elucidate the mechanisms underlying CAF-induced HOTAIR activation, we used ChIP technology to identify the genomic locations bound by SMADs in cancer cells before and after co-culture with CAF-CM. The ChIP results indicated that CAFs stimulate the direct binding of SMAD2, 3, and 4 to the promoter region of HOTAIR (Fig. 4c), and a promoter analysis using JASPAR and PROMO indicated several candidate SMAD binding sites in the promoter region of HOTAIR (Fig. 4d). The relative profile score threshold set as 80% in JASPAR and the maximum matrix dissimilarity rate set as 15% in PROMO [28, 29]. Accordingly, the deletion of predicted binding sites (Mut-1 and Mut-2) within the HOTAIR promoter region decreased SMAD2/3/4 induced luciferase activity in MCF-7 and MDA-MB-231 cells, whereas the deletion of the remaining mutant binding sites did not result in significant differences compared with control group (Fig. 4e). Therefore, we concluded that SMADs directly bind the promoter site of HOTAIR located between nucleotides -386 and -398, -440 and -452 (the genome coordinates: chr12: 53975342-53975354, chr12: 53975396-53975408), and consequently induced the transactivation of HOTAIR.

Based on these results, we elucidated that CAFs transactivated HOTAIR via the secretion TGF-β1. Furthermore, we identified HOTAIR as a direct transcriptional target of SMAD2/3/4.

### CAFs transactivated HOTAIR expression to induce EMT by targeting CDK5 signaling

**Fig. 5:**
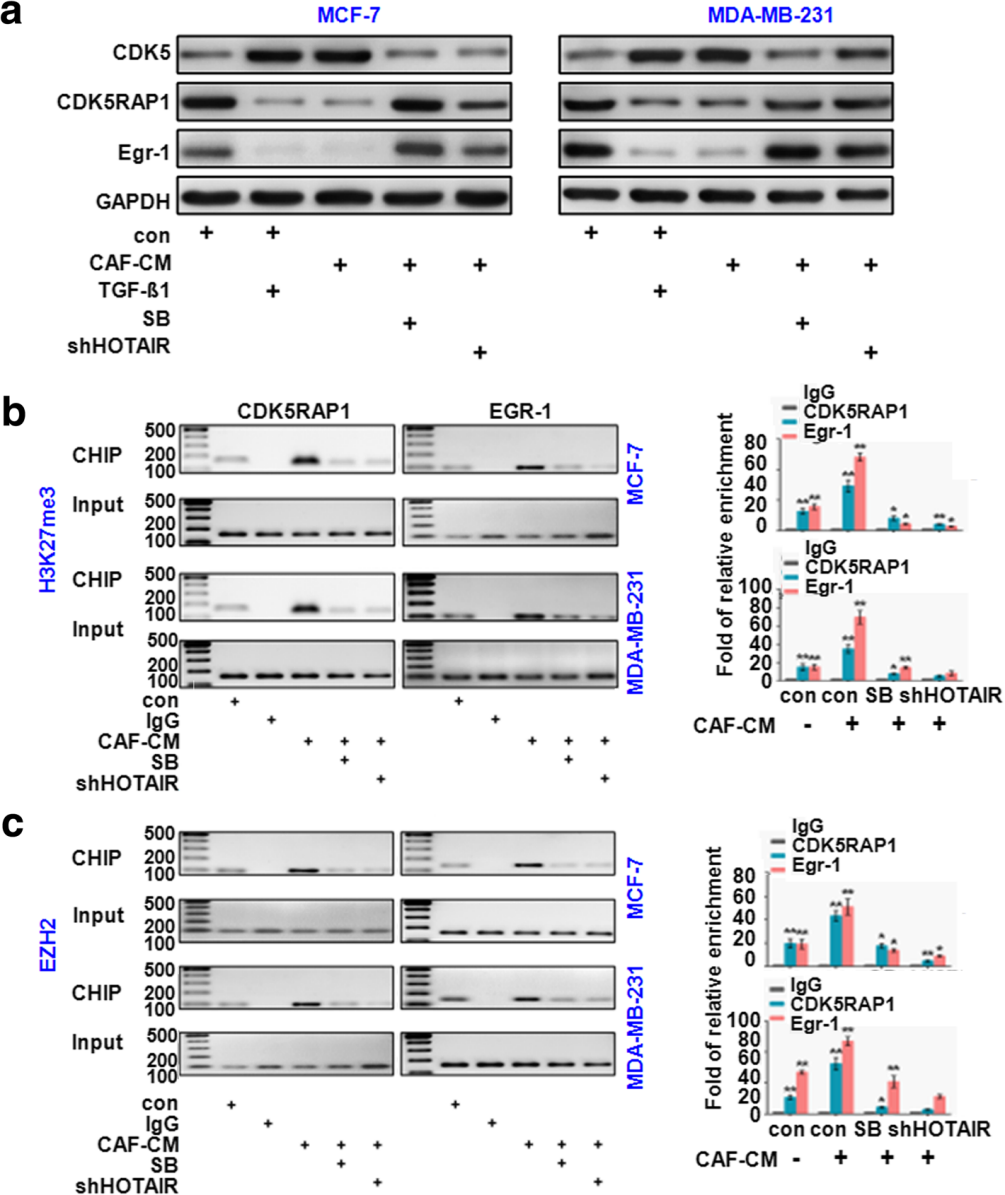
CAFs transactivated HOTAIR expression to induce EMT by targeting CDK5 signaling. **a** Western blotting indicated that CDK5 expression was significantly increased in recombinant TGF-β1- or CAF-CM treated cells, whereas the EGR-1 and CDK5RAP1 expression levels were greatly decreased. **b** The histone methylation status of CDK5 signaling was determined with a CHIP assay, which indicated that H3K27me3 was enriched at the promoter of EGR-1 and CDK5RAP1 in CAF-CM-treated MDA-MB-231 and MCF-7 cells, whereas the depletion of HOTAIR blocked this function of CAFs. The results are presented as the fold-change over the control. **c** ChIP analysis revealed that elevated EZH2 was recruited to the EGR-1 and CDK5RAP1 promoter in MDA-MB-231 and MCF-7 cells. All experiments were repeated three times. *P < 0.05 vs. negative control

We previously reported that the active form of CDK5 kinase was responsible for tumor metastasis. Consistent with our previous study, CDK5 protein expression was significantly increased in recombinant TGF-β1- or CAF-CM treated cells, whereas the levels of EGR-1 and CDK5RAP1 were greatly decreased (Fig. 5a). These findings suggest that the activation of CDK5 was essential for CAF-induced EMT. To investigate the epigenetic events that were involved in the activation of CDK5 in breast cancer, we first examined the histone methylation status using ChIP assays (Fig. 5b). Profiling histone methylation marks on the CDK5RAP1 and EGR-1 promoter by qChIP showed that H3K27me3 is highly enriched at the promoter of both genes in CAF-CM treated MDA-MB-231 and MCF-7 cells compared with the control cells. Whereas the depletion of HOTAIR rescued the effect of CAFs, the H3K27me3 levels at the CDK5RAP1 and EGR-1 promoter were remarkably reduced. Furthermore, the recruitment of EZH2 to the CDK5RAP1 and EGR-1 promoter was measured, which indicated that the level of PRC2 components at the CDK5RAP1 and EGR-1 promoter was significantly decreased (Fig. 5c). Based on these data, we demonstrated that the activation of CDK5 was required for CAF-mediated EMT. Specifically, CAFs stimulated CDK5 expression by H3K27 trimethylation on the promoter of CDK5RAP1 and EGR-1. CAFs transactivated HOTAIR expression to induce EMT by targeting CDK5 signaling.

### Knock-down HOTAIR inhibited CAF-induced tumor growth and lung metastasis in vivo

**Fig. 6:**
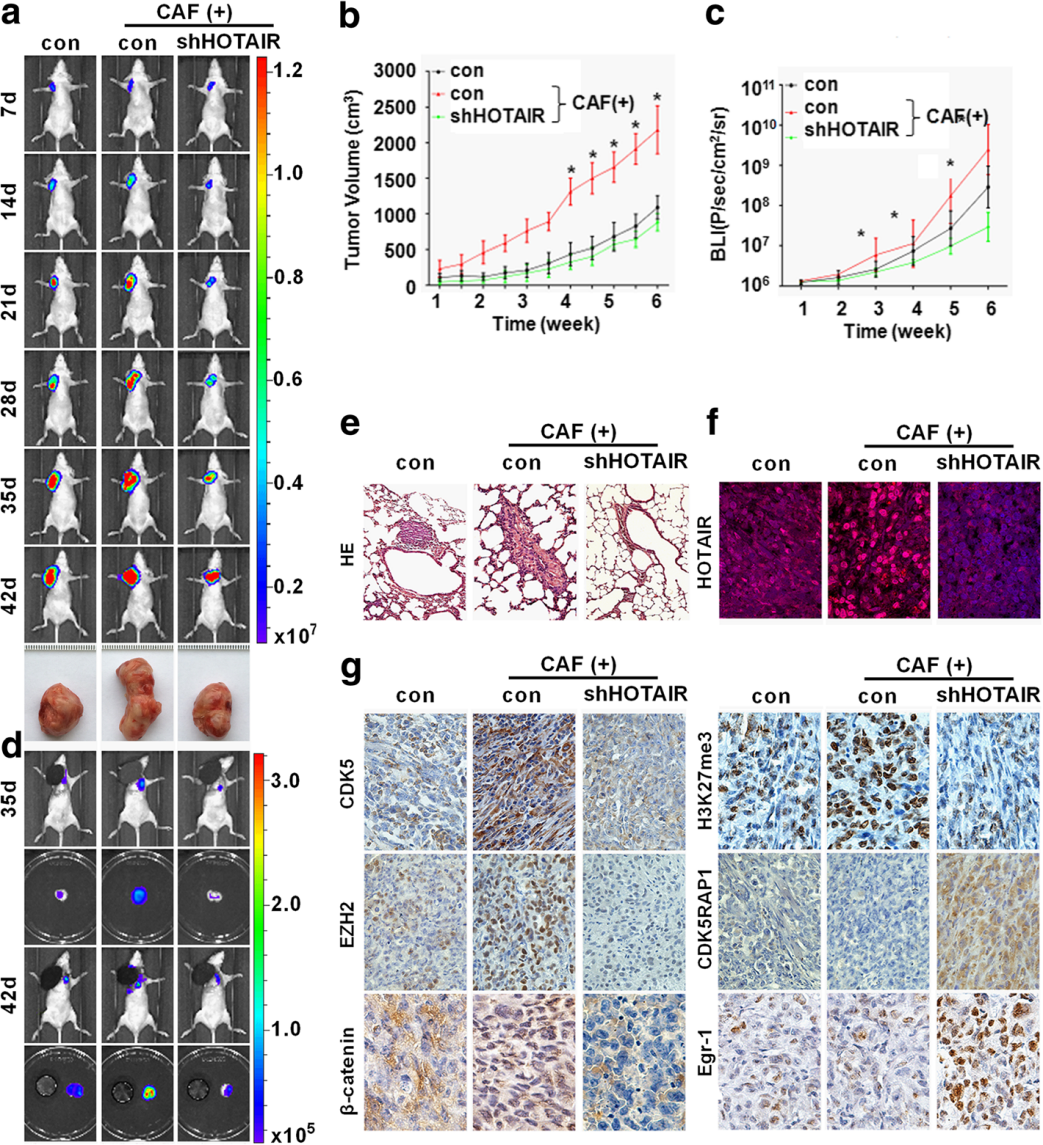
The depletion of HOTAIR suppressed CAF-induced tumor growth and metastasis in vivo. **a** Representative pseudocolor bioluminescence images of tumor size were obtained on days 7, 14, 21, 28, 35, and 42. Mice bearing orthotopic tumors of MDA-MB-231-Luc cells were transfected with control shRNA or shHOTAIR. Each group included eight mice (*n* = 8). Primary tumor volumes (**b**) and representative bioluminescence images (BLI) (**c**) were evaluated. **d** Lung metastasis was detected by bioluminescent imaging on days 35 and 42. **e** Representative photomicrographs of H&E-stained lung tissues. **f** The expression of HOTAIR was assessed by FISH. **g** Representative images of the immunohistochemical staining of CDK5, EZH2, ß-catenin, H3K27me3, CDK5RAP1 and EGR-1 in tissues. Scale bars in all images: 15 μm

To study the epigenetic mechanism of CAF-induced tumor growth and metastasis in vivo, an MDA-MB-231 orthotopic tumor transplantation model was established in nude mice. Bioluminescence imaging was monitored weekly and indicated that all of the four CAF populations remarkably promoted primary tumor growth, whereas shHOTAIR treatment markedly reduced tumor volume (Fig. 6a-c, Additional file 1: Figure S6). Most importantly, CAFs resulted in dramatic lung metastasis with extensive tumor foci compared to control cells, as determined by quantitative bioluminescence images (Fig. 6d). However, this metastatic phenomenon was attenuated when HOTAIR was knocked down, and H&E staining further confirmed these results (Fig. 6e). Compared to the control group, CAFs enhanced the nuclear staining of HOTAIR, as revealed by FISH, which further confirmed our in vitro study (Fig. 6f). Moreover, immunohistochemistry staining showed the downregulation of nuclear β-catenin and reductions in p-SMAD2-, p-SMAD3-, EZH2- and H3K27me3- positive cells in the shHOTAIR group (Fig. 6g). Taken together, these findings indicate that shHOTAIR treatment efficiently blocked the crosstalk between CAFs and tumor cells, thereby impairing CAF-induced EMT. Collectively, these experiments supported the notion that the secretion TGF-β1 by CAFs activated the TGF-β1/SMAD pathway in breast cancer cells, resulting in the up-regulation of HOTAIR transcription and histone modification of the CDK5 signaling pathway, thereby promoting breast cancer progression and metastasis.

### Aberrant HOTAIR correlated with a poor prognosis and metastasis in breast cancer

**Fig. 7:**
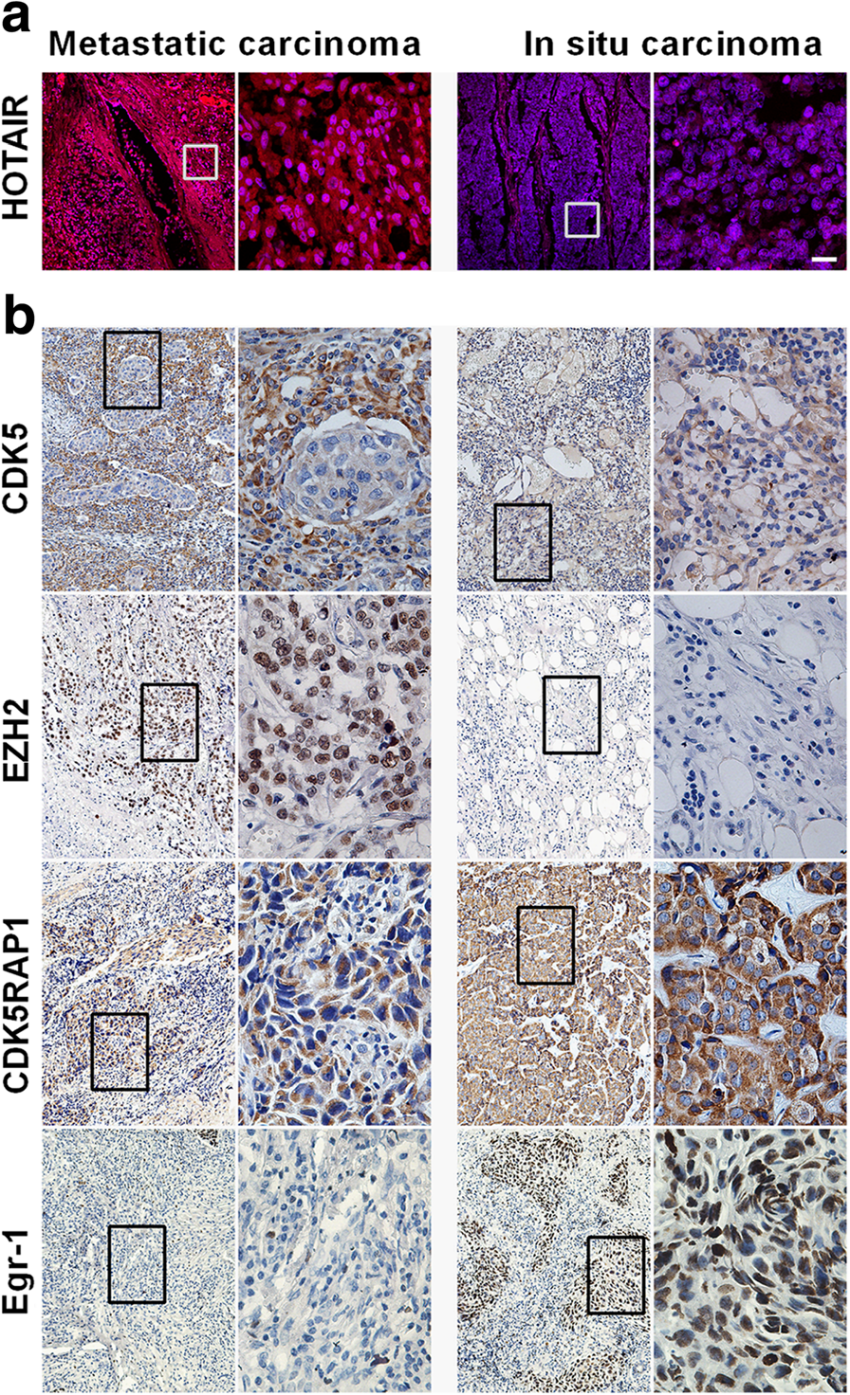
Overexpressed HOTAIR correlated with poor prognosis in breast cancer. **a** HOTAIR expression was analyzed by FISH. Scale bars in all images: 15 μm. **b** Immunohistochemistry staining detected EZH2, CDK5, CDK5RAP1 and EGR-1 expression in 59 breast carcinoma samples (39 with lymph node metastasis, 19 in situ carcinoma)

To gain a further insight of the role of CAFs in the clinical setting, we collected 59 breast carcinoma samples and examined markers of CAFs FAP-α and α-SMA by immunohistochemistry. We found that the expression levels of FAP-α and α-SMA were significantly up-regulated in invasive breast carcinoma (97.5%, 38 in 39), compared with human ductal carcinoma in situ (DCIS, 33.3%, 7 of 20), suggesting that CAFs facilitated tumor metastasis in patients with breast cancer (Additional file 1: Figure S7). Compared to DCIS, HOTAIR expression and nuclear staining were higher in metastatic breast carcinoma, as revealed by FISH images, which indicated that the upregulation of HOTAIR strongly correlated with tumor metastasis (Fig. 7a). Furthermore, the expression levels of EZH2 and CDK5 exhibited similar patterns, which further confirmed our in vitro and in vivo studies (Fig. 7b).

## Discussion

**Fig. 8:**
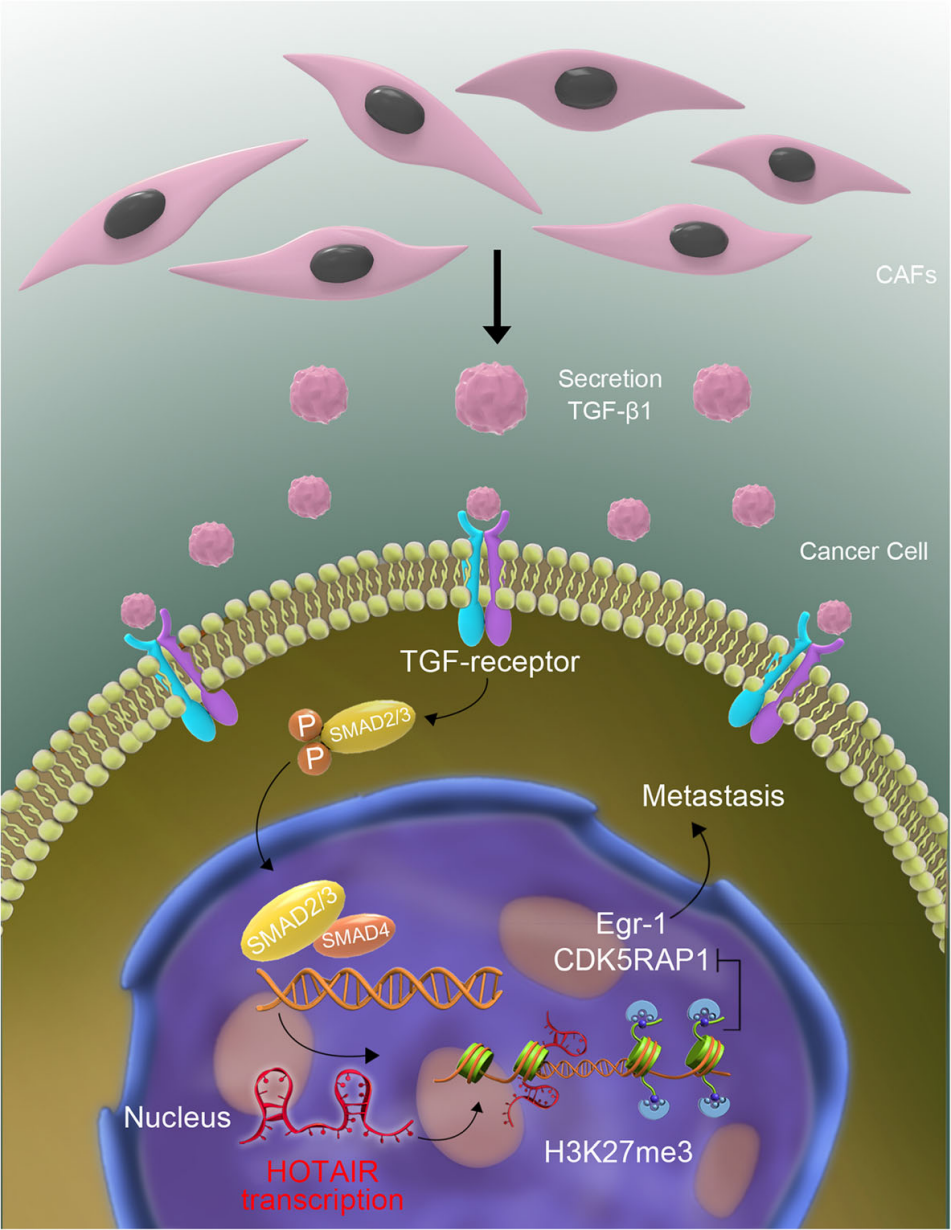
The proposed model of the paracrine and epigenetic mechanism of CAFs induced EMT and metastasis in breast cancer. CAFs secreted high levels of TGF-β1, resulting in the transactivation of HOTAIR in breast cancer cells, consequently enhanced metastasis

Based on our results, we propose a model of the CAF-mediated upregulation of HOTAIR transcription in breast cancer cells that promotes invasion and metastasis via the secretion of TGF-β1 (Fig. 8). The hyperactivation of TGF-β1 signaling clearly contributes to tumor metastasis and the metabolic reprogramming and activation of CAFs [30, 31]. Moreover, TGF-β1 has been linked to poor clinical outcome and is required for the CAF-mediated initiation of efficient metastasis [26, 32, 33]. Consistent with these results, our data further indicate that the key pro-metastatic function of CAFs depends on TGF-β1 secretion (Fig. 2). Specifically, blocking TGF-β1 secretion with SB or PFDefficiently abrogated CAF-mediated metastasis. Our study demonstrated that TGF-β1 is a crucial mediator of the cross-talk between stromal and cancer cells within the tumor microenvironment.

Non-coding RNAs have been added to the growing list of gene regulators that contribute to the epigenetic regulation of gene expression [34]. Specifically, mounting evidence has revealed that the up-regulation of HOTAIR contributed to drug resistance and tumor metastasis in various types of solid tumors. In this study, our experiment demonstrated that CAFs upregualted HOTAIR expression via TGF-β1 secretion, supporting its importance in breast cancer progression (Fig. 3b-f). Most importantly, SMAD2/3/4 directly bound the promoter site of HOTAIR, as revealed by CHIP and luciferase reporter assay, suggesting that HOTAIR was a transcriptional target of SMAD2/3/4 (Fig. 4c and e). These findings suggested that the secretion of TGF-β1 by CAFs clearly led to the transactivation of HOTAIR expression. And the breast cancer microenvironment plays a key role in maintaining HOTAIR expression in cancer cells. Our studies provide a strong body of evidence to support that the modulation of epigenetic control and targeted alterations of HOTAIR represent novel therapeutic targets in breast cancer.

Extensive reports have identified several microRNA induced by CAFs (e.g., miR-26b, miR-148a and miR-133b) that were invovled in tumor progression [35–37]. Specifically, our previous study verified that CAFs impaired taxol efficacy by activating miR-21 expression. To the best of our knowledge, this study is the first to describe that the CAF-mediated expression of long non-coding RNA led to enhanced cell migration and invasion.

We previously reported that the active form of CDK5 kinase was responsible for tumor metastasis. However, the molecular mechanism of CDK5 activation has not been fully elucidated. In cancer, gene expression profiles revealed a loss of tumor suppressor gene expression due to aberrant histone modification. Specifically, Kumar et al. reported that H3 hyperacetylation at the promoter of CDK5 was a key mechanism underlying cocaine-induced plasticity in the striatum [38]. In the present study, we proved that CAFs mediated the transcription of HOTAIR, leading to a strong increase in the H3K27-mediated trimethylation of the CDK5RAP1 and EGR-1 promoters, thereby activating CDK5 expression and EMT.

Although our study focuses on the TGF-β1, CAFs may also secrete a cocktail of additional pro-metastatic factors, such as IL-6 and IGF-II, to contribute to cancer progression [39, 40]. Moreover, additional crucial lncRNAs involved in the development of breast cancer remain to be investigated.

## Conclusions

CAFs may orchestrate multiple non-coding RNA and signaling pathways to control several biological processes in a paracrine manner during breast cancer progression. We reported here that secretion of TGF-β1 by CAFs was a crucial mediator of the cross-talk between stromal cells and cancer cells to promote EMT and metastasis in breast cancer. Our study revealed a novel epigenetic mechanism underlying CAF-induced tumor growth and metastasis, the TGF-ß1/HOTAIR axis controls the development and progression of breast cancer, which supports the pursuit of these molecules as targets of breast cancer treatment.

## Additional file

Additional file 1: Figure S1-S7. Showing supplementary results. (DOCX 8533 kb)

## Abbreviations

BC: Breast cancer
CAFs: Carcinoma associated fibroblasts
DMEM: Dulbecco’s modified Eagle’s medium
EMT: Epithelial–mesenchymal transition
FBS: Fetal bovine serum
HOTAIR: HOX transcript antisense RNA
PBS: Phosphate-buffered saline
PCR: Polymerase chain reaction
PFD: Pirfenidone
SB: SB431542

## References

[1] Weigelt B, Peterse JL, van’t Veer LJ (2005). Breast cancer metastasis: markers and models. Nat Rev Cancer.

[2] Daniel CW, Deome KB (1965). Growth of mouse mammary gland in vivo after monolayer culture. Science.

[3] Arendt LM, Rudnick JA, Keller PJ, Kuperwasser C (2010). Stroma in breast development and disease. Semin Cell Dev Biol.

[4] Yang L, Achreja A, Yeung TL, Mangala LS, Jiang D, Han C, Baddour J, Marini JC, Ni J, Nakahara R (2016). Targeting stromal glutamine synthetase in tumors disrupts tumor microenvironment-regulated cancer cell growth. Cell Metab.

[5] Erez N, Truitt M, Olson P, Arron ST, Hanahan D (2010). Cancer-associated fibroblasts are activated in incipient neoplasia to orchestrate tumor-promoting inflammation in an NF-kappaB-dependent manner. Cancer Cell.

[6] Quante M, Tu SP, Tomita H, Gonda T, Wang SS, Takashi S, Baik GH, Shibata W, Diprete B, Betz KS (2011). Bone marrow-derived myofibroblasts contribute to the mesenchymal stem cell niche and promote tumor growth. Cancer Cell.

[7] Servais C, Erez N (2013). From sentinel cells to inflammatory culprits: cancer-associated fibroblasts in tumour-related inflammation. J Pathol.

[8] Mueller MM, Fusenig NE (2004). Friends or foes - bipolar effects of the tumour stroma in cancer. Nat Rev Cancer.

[9] Gascard P, Tlsty TD (2016). Carcinoma-associated fibroblasts: orchestrating the composition of malignancy. Genes Dev.

[10] Ren Y, Zhou X, Liu X, Jia HH, Zhao XH, Wang QX, Han L, Song X, Zhu ZY, Sun T (2016). Reprogramming carcinoma associated fibroblasts by AC1MMYR2 impedes tumor metastasis and improves chemotherapy efficacy. Cancer Lett.

[11] Sarkar D, Leung EY, Baguley BC, Finlay GJ, Askarian-Amiri ME (2015). Epigenetic regulation in human melanoma: past and future. Epigenetics.

[12] Wu L, Murat P, Matak-Vinkovic D, Murrell A, Balasubramanian S (2013). Binding interactions between long noncoding RNA HOTAIR and PRC2 proteins. Biochemistry.

[13] Teschendorff AE, Lee SH, Jones A, Fiegl H, Kalwa M, Wagner W, Chindera K, Evans I, Dubeau L, Orjalo A (2015). HOTAIR and its surrogate DNA methylation signature indicate carboplatin resistance in ovarian cancer. Genome Med.

[14] Xue X, Yang YA, Zhang A, Fong KW, Kim J, Song B, Li S, Zhao JC, Yu J (2016). LncRNA HOTAIR enhances ER signaling and confers tamoxifen resistance in breast cancer. Oncogene.

[15] Malek E, Jagannathan S, Driscoll JJ (2014). Correlation of long non-coding RNA expression with metastasis, drug resistance and clinical outcome in cancer. Oncotarget.

[16] Padua Alves C, Fonseca AS, Muys BR, de Barros ELBR, Burger MC, de Souza JE, Valente V, Zago MA, Silva WA (2013). Brief report: the lincRNA Hotair is required for epithelial-to-mesenchymal transition and stemness maintenance of cancer cell lines. Stem Cells.

[17] Gupta RA, Shah N, Wang KC, Kim J, Horlings HM, Wong DJ, Tsai MC, Hung T, Argani P, Rinn JL (2010). Long non-coding RNA HOTAIR reprograms chromatin state to promote cancer metastasis. Nature.

[18] Yang Z, Zhou L, Wu LM, Lai MC, Xie HY, Zhang F, Zheng SS (2011). Overexpression of long non-coding RNA HOTAIR predicts tumor recurrence in hepatocellular carcinoma patients following liver transplantation. Ann Surg Oncol.

[19] Kim K, Jutooru I, Chadalapaka G, Johnson G, Frank J, Burghardt R, Kim S, Safe S (2013). HOTAIR is a negative prognostic factor and exhibits pro-oncogenic activity in pancreatic cancer. Oncogene.

[20] Cao L, Zhou J, Zhang J, Wu S, Yang X, Zhao X, Li H, Luo M, Yu Q, Lin G (2015). Cyclin-dependent kinase 5 decreases in gastric cancer and its nuclear accumulation suppresses gastric tumorigenesis. Clin Cancer Res.

[21] Quintavalle M, Elia L, Price JH, Heynen-Genel S, Courtneidge SA (2011). A cell-based high-content screening assay reveals activators and inhibitors of cancer cell invasion. Sci Signal.

[22] Mak GW, Chan MM, Leong VY, Lee JM, Yau TO, Ng IO, Ching YP (2011). Overexpression of a novel activator of PAK4, the CDK5 kinase-associated protein CDK5RAP3, promotes hepatocellular carcinoma metastasis. Cancer Res.

[23] Ren Y, Zhou X, Yang JJ, Liu X, Zhao XH, Wang QX, Han L, Song X, Zhu ZY, Tian WP (2015). AC1MMYR2 impairs high dose paclitaxel-induced tumor metastasis by targeting miR-21/CDK5 axis. Cancer Lett.

[24] Liang Q, Li L, Zhang J, Lei Y, Wang L, Liu DX, Feng J, Hou P, Yao R, Zhang Y (2013). CDK5 is essential for TGF-beta1-induced epithelial-mesenchymal transition and breast cancer progression. Sci Rep.

[25] Leshchenko VV, Kuo PY, Shaknovich R, Yang DT, Gellen T, Petrich A, Yu Y, Remache Y, Weniger MA, Rafiq S (2010). Genomewide DNA methylation analysis reveals novel targets for drug development in mantle cell lymphoma. Blood.

[26] Barcellos-de-Souza P, Comito G, Pons-Segura C, Taddei ML, Gori V, Becherucci V, Bambi F, Margheri F, Laurenzana A, Del Rosso M (2016). Mesenchymal stem cells are recruited and activated into carcinoma-associated fibroblasts by prostate cancer microenvironment-derived TGF-beta1. Stem Cells.

[27] Derynck R, Zhang YE (2003). Smad-dependent and Smad-independent pathways in TGF-beta family signalling. Nature.

[28] Messeguer X, Escudero R, Farré D, Núñez O, Martínez J, Albà MM (2002). PROMO: detection of known transcription regulatory elements using species-tailored searches. Bioinformatics.

[29] Sandelin A, Alkema W, Engström P, Wasserman WW, Lenhard B (2004). JASPAR: an open-access database for eukaryotic transcription factor binding profiles. Nucleic Acids Res.

[30] Yang J, Lu Y, Lin YY, Zheng ZY, Fang JH, He S, Zhuang SM (2016). Vascular mimicry formation is promoted by paracrine TGF-beta and SDF1 of cancer-associated fibroblasts and inhibited by miR-101 in hepatocellular carcinoma. Cancer Lett.

[31] Guido C, Whitaker-Menezes D, Capparelli C, Balliet R, Lin Z, Pestell RG, Howell A, Aquila S, Ando S, Martinez-Outschoorn U (2012). Metabolic reprogramming of cancer-associated fibroblasts by TGF-beta drives tumor growth: connecting TGF-beta signaling with “Warburg-like” cancer metabolism and L-lactate production. Cell Cycle.

[32] Calon A, Espinet E, Palomo-Ponce S, Tauriello DV, Iglesias M, Cespedes MV, Sevillano M, Nadal C, Jung P, Zhang XH (2012). Dependency of colorectal cancer on a TGF-beta-driven program in stromal cells for metastasis initiation. Cancer Cell.

[33] Calon A, Lonardo E, Berenguer-Llergo A, Espinet E, Hernando-Momblona X, Iglesias M, Sevillano M, Palomo-Ponce S, Tauriello DV, Byrom D (2015). Stromal gene expression defines poor-prognosis subtypes in colorectal cancer. Nat Genet.

[34] Hauptman N, Glavac D (2013). Long non-coding RNA in cancer. Int J Mol Sci.

[35] Verghese ET, Drury R, Green CA, Holliday DL, Lu X, Nash C, Speirs V, Thorne JL, Thygesen HH, Zougman A (2013). MiR-26b is down-regulated in carcinoma-associated fibroblasts from ER-positive breast cancers leading to enhanced cell migration and invasion. J Pathol.

[36] Min A, Zhu C, Peng S, Shuai C, Sun L, Han Y, Qian Y, Gao S, Su T (2016). Downregulation of Microrna-148a in cancer-associated fibroblasts from oral cancer promotes cancer cell migration and invasion by targeting Wnt10b. J Biochem Mol Toxicol.

[37] Doldi V, Callari M, Giannoni E, D'Aiuto F, Maffezzini M, Valdagni R, Chiarugi P, Gandellini P, Zaffaroni N (2015). Integrated gene and miRNA expression analysis of prostate cancer associated fibroblasts supports a prominent role for interleukin-6 in fibroblast activation. Oncotarget.

[38] Kumar A, Choi KH, Renthal W, Tsankova NM, Theobald DE, Truong HT, Russo SJ, Laplant Q, Sasaki TS, Whistler KN (2005). Chromatin remodeling is a key mechanism underlying cocaine-induced plasticity in striatum. Neuron.

[39] Osuala KO, Sameni M, Shah S, Aggarwal N, Simonait ML, Franco OE, Hong Y, Hayward SW, Behbod F, Mattingly RR (2015). Il-6 signaling between ductal carcinoma in situ cells and carcinoma-associated fibroblasts mediates tumor cell growth and migration. BMC Cancer.

[40] Chen WJ, Ho CC, Chang YL, Chen HY, Lin CA, Ling TY, Yu SL, Yuan SS, Chen YJ, Lin CY (2014). Cancer-associated fibroblasts regulate the plasticity of lung cancer stemness via paracrine signalling. Nat Commun.

