# Supplementary material for: Paracrine and epigenetic control of CAF-induced metastasis: the role of HOTAIR stimulated by TGF-ß1 secretion
Source: Mol Cancer. 2018 Jan 11;17:5. doi: 10.1186/s12943-018-0758-4 (PMC5765658; doi:10.1186/s12943-018-0758-4)
Supplement: Additional file 1: Figure S1-S7. — Showing supplementary results. (DOCX 8533 kb) [file 12943_2018_758_MOESM1_ESM.docx]

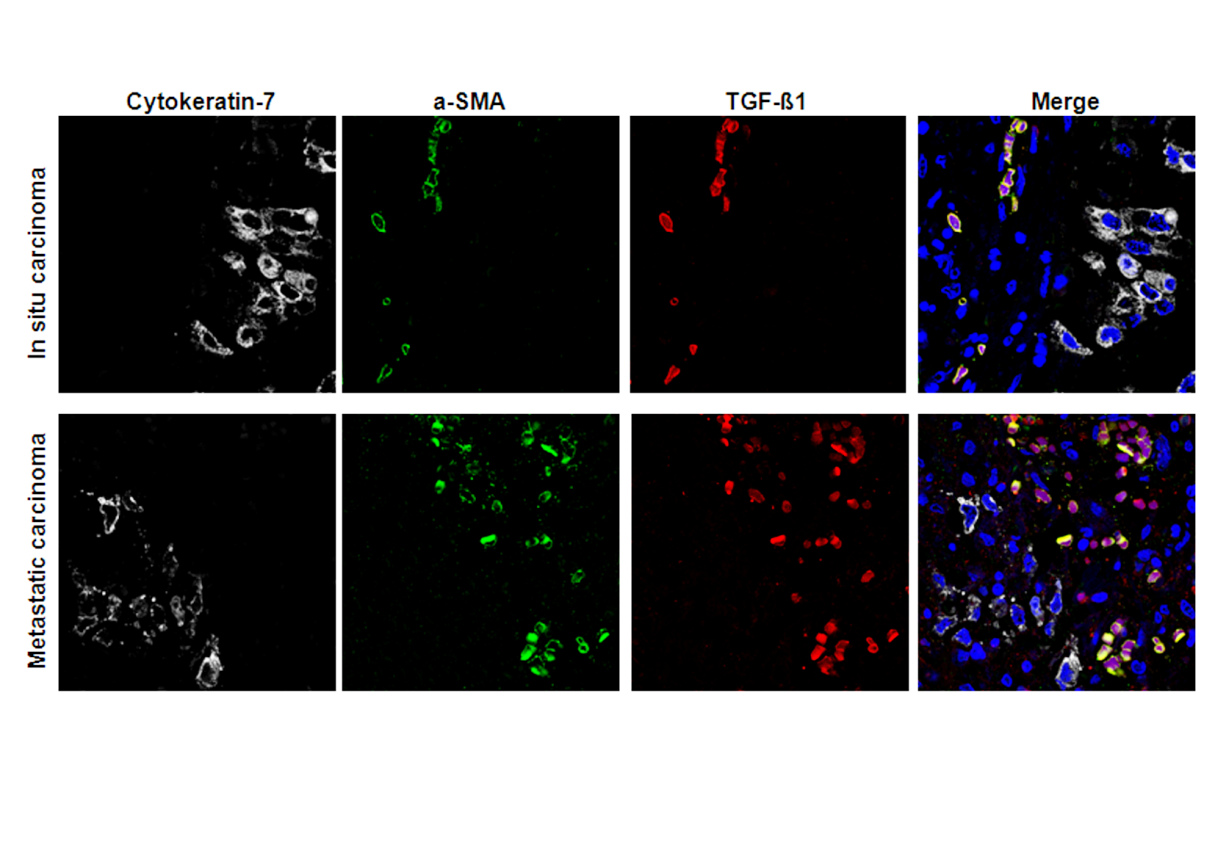


**Fig.S1** Immunofluorescent staining with antibodies against α-SMA, cytokeratin-7 and TGF-ß1 form invasive breast carcinoma patients and ductal carcinoma in situ patients, respectively.


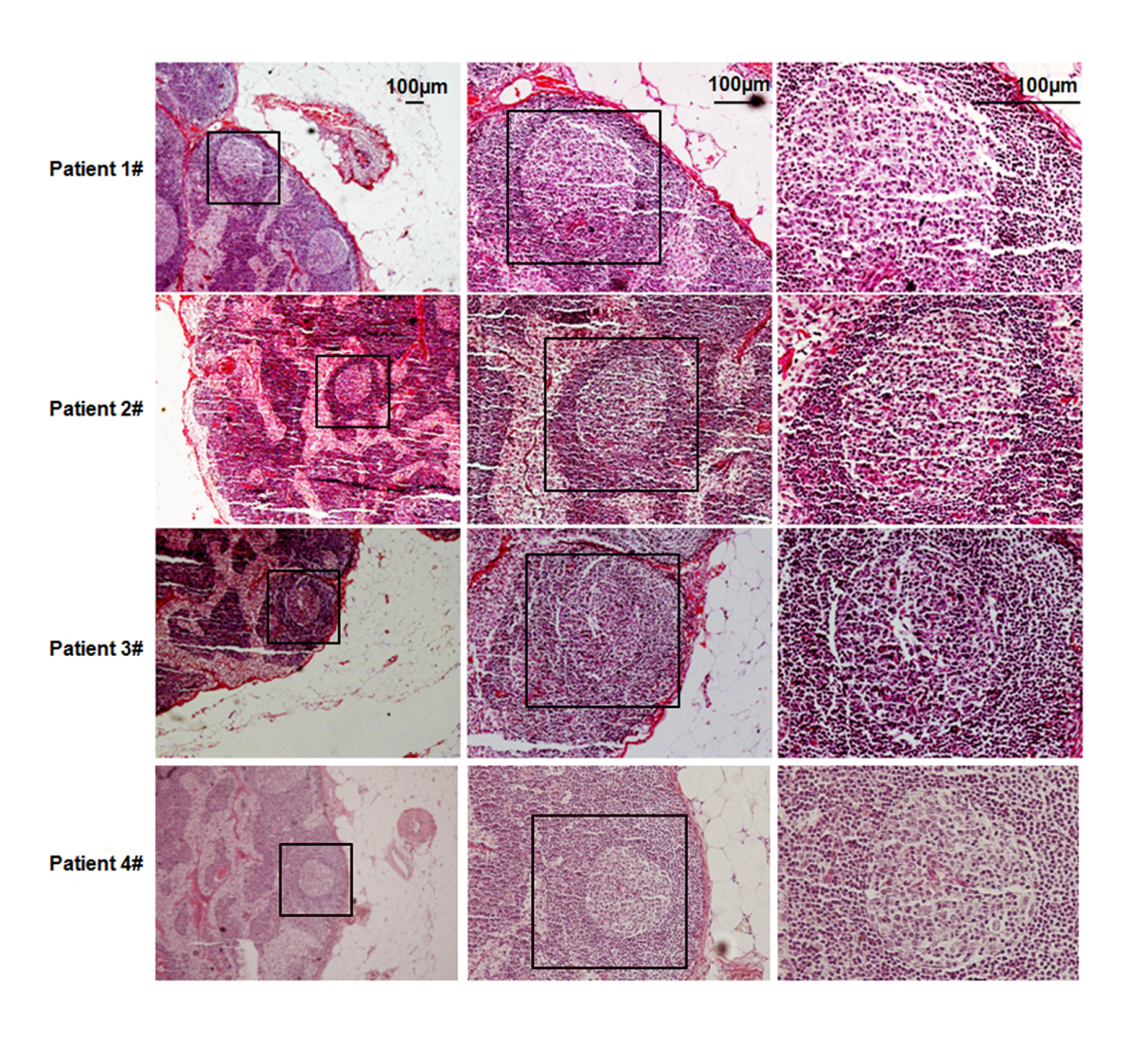


**Fig.S2** H&E staining of four invasive breast carcinoma samples.

.


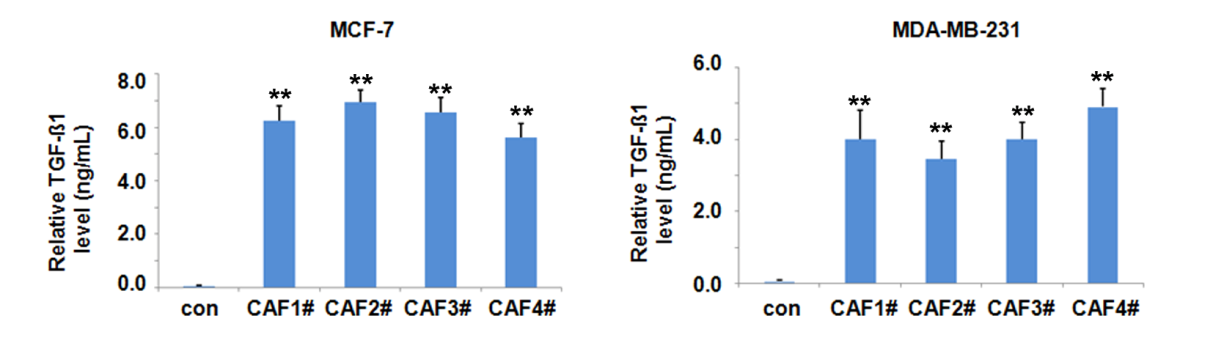


**Fig.S3** Secretion of TGF-β1 in four different CAF populations. The amount of TGF-β1 released into the cell culture supernatant of cells treated with various CAF-CM was determined by ELISA.


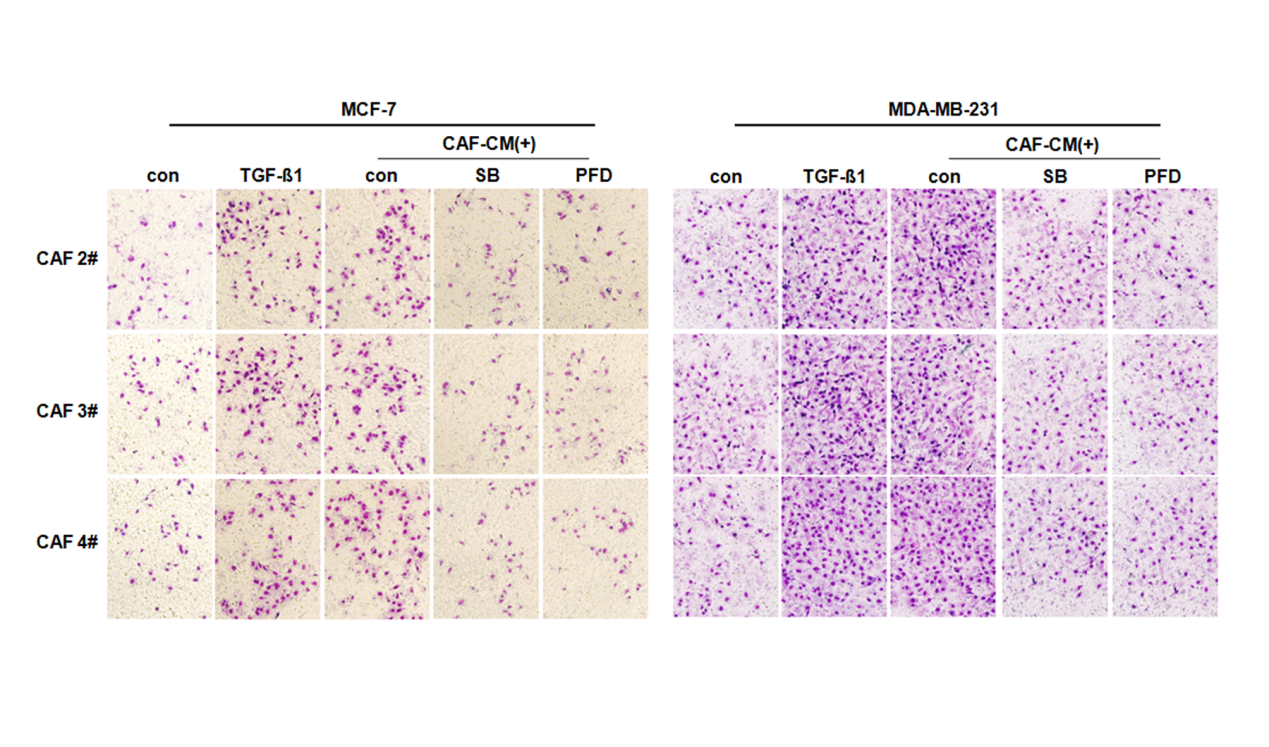


**Fig.S4** Promotion of cell invasive ability in four different CAF populations. CAF-CM treatment enhanced cell invasive ability, SB or PFD treatment impaired the CAF-mediated increase in invading cancer cells, as indicated by the Transwell assay. The result of CAF1# was shown in Fig. 2b of the original manuscript.


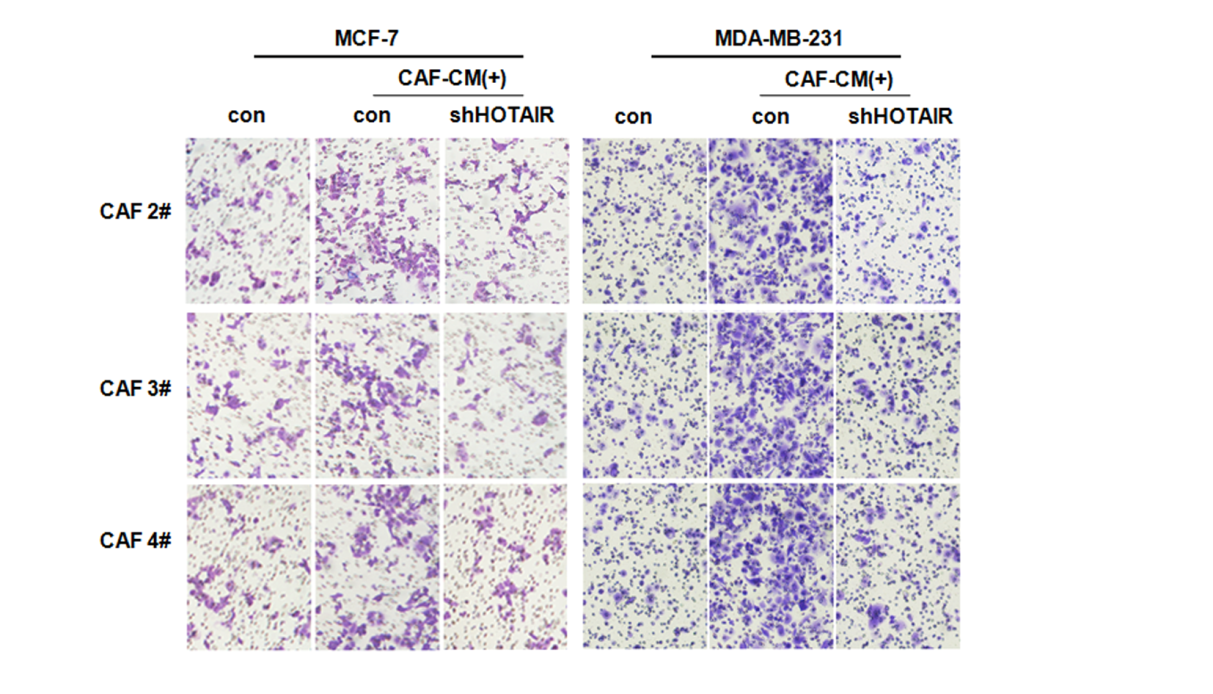
**Fig.S5** The depletion of HOTAIR decreased the number of invaded cells induced by CAF-CM, as indicated by the Transwell assay. The result of CAF1# was shown in Fig. 3f of the original manuscript.


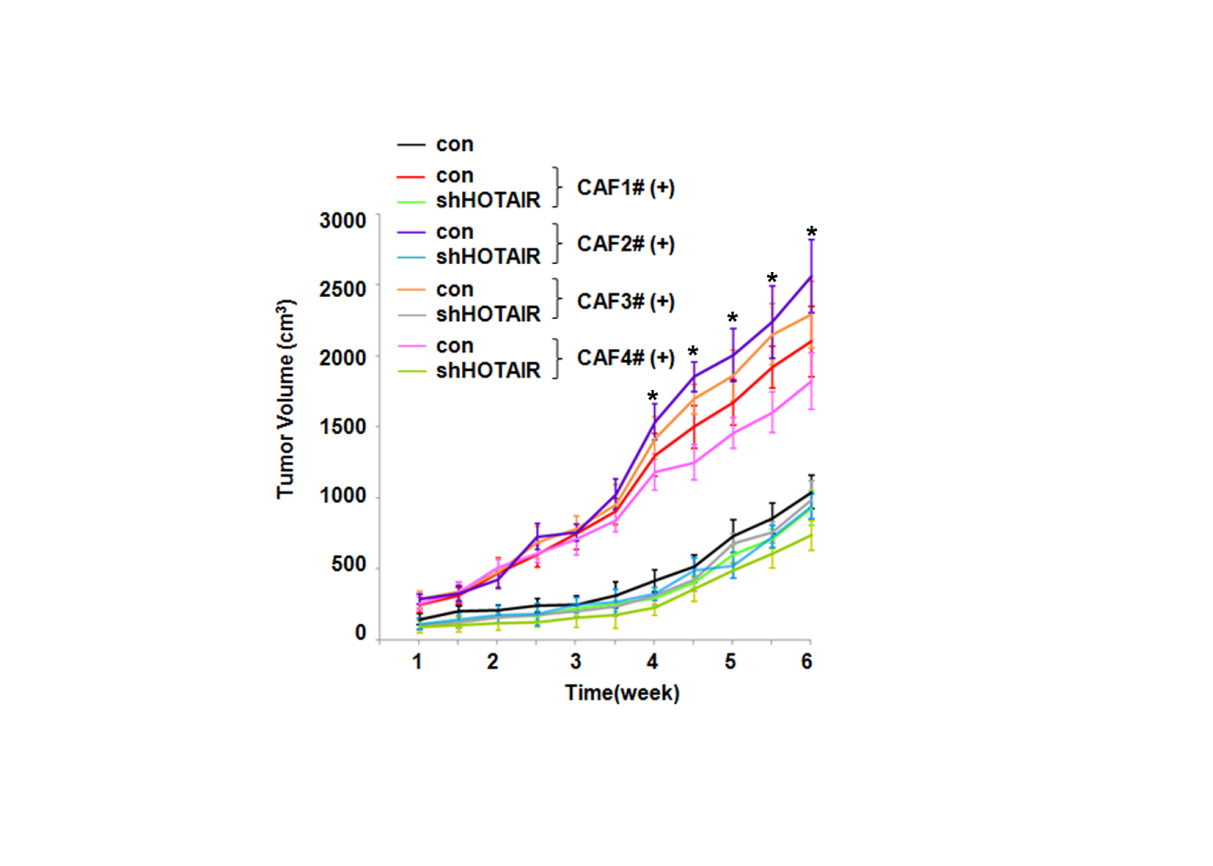


**Fig.S6** Tumor-promoting ability in four different CAF populations. Mice bearing orthotopic tumors of MDA-MB-231 cells were transfected with control shRNA or shHOTAIR. Primary tumor volumes were evaluated. All of the four CAF populations remarkably promoted primary tumor growth. The depletion of HOTAIR suppressed CAF-induced tumor growth and metastasis in vivo.


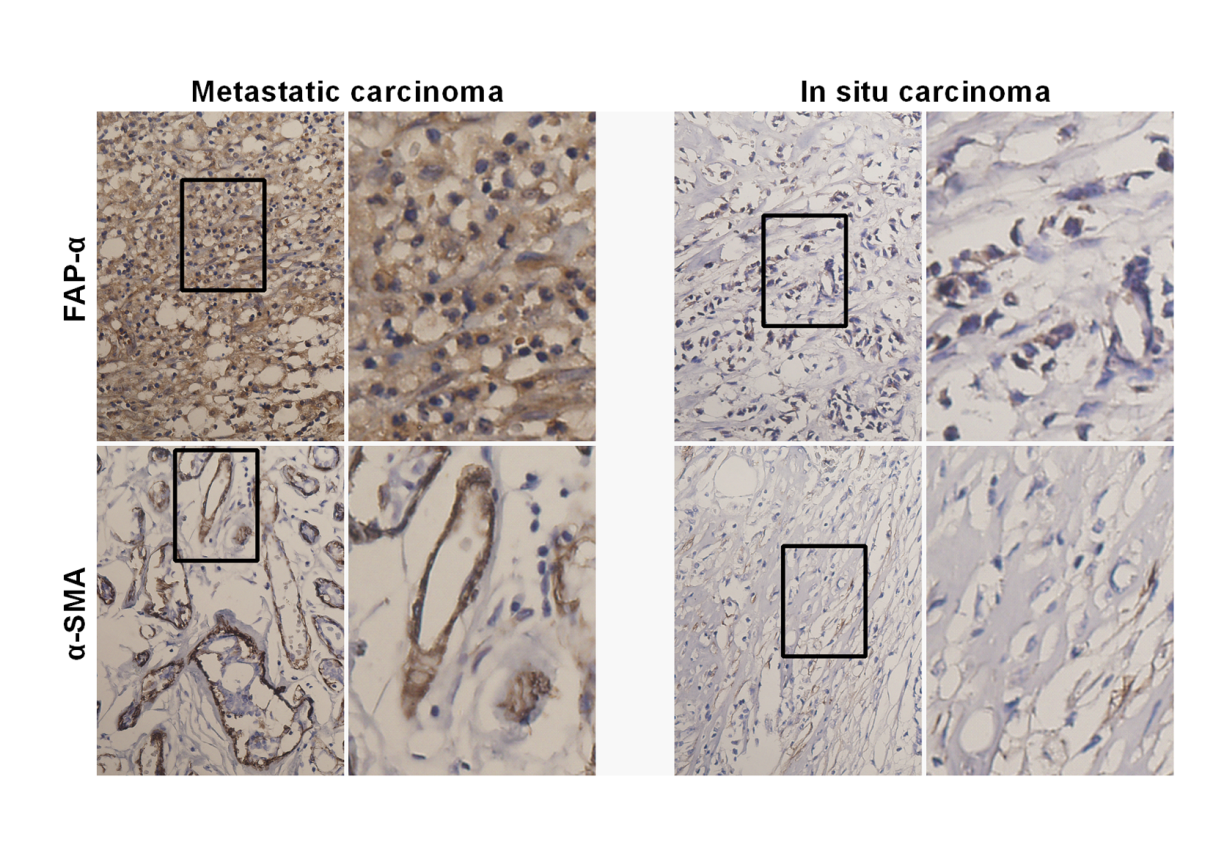


**Fig.S7** High expression of FAP-α and α-SMA associated with tumor metastasis. FAP-α and α-SMA was detected by in-situ hybridization and immunohistochemistry in lymph node metastasis and in situ carcinoma, respectively.
